# Tri-layer Tympanoplasty as a New Technique in High-risk Tympanic Membrane Perforations

**Published:** 2019-09

**Authors:** Fevzi Solmaz, Davut Akduman, Mehmet Haksever

**Affiliations:** 1 *Department of Otorhinolaryngology, Health Science University, Bursa Yuksek Ihtisas Training and Research Hospital,16800 Yıldırım, Bursa, Turkey.*; 2 *Department of Otorhinolaryngology, Duzce University Hospital, Duzce, Turkey.*

**Keywords:** Cartilage tympanoplasty, Chronic otitis media, Tri-layer tympanoplasty, Temporal fascia, Tragal cartilage.

## Abstract

**Introduction:**

The eradication of the middle ear disease is mentioned as the fundamental principle of tympanoplasty. The presence of some factors related to patient or disease itself forces the physician to classify the chronic ear disease as high-risk perforations. The aim of this study was to present a tri-layer tympanoplasty technique and its otological and audiological outcomes in the ears with high-risk perforations.

**Materials and Methods:**

This retrospective study was carried out on a total of 46 eligible ears that had chronic otitis media with high-risk perforations. Preoperatively, 17, 15, and 14 ears were reported with Sade classification grade 4 pars tensa retraction (Group 1), total or near-total tympanic membrane perforation (Group 2), and a history of ear surgery (Group 3), respectively. All the cases had tympanoplasty using the tri-layer technique at a tertiary center during 2008 and 2014. A review of the patients’ chart showed that 46 patients underwent tri-layer tympanoplasty. Regarding the audiological outcomes, the comparison of pre- and post-operative results revealed mean air conduction level and mean air-bone gap (ABG) of 4 different frequencies in dB according to a new standardized format for reporting hearing outcome in clinical trials.

**Results:**

The mean value of the follow-up period was reported as 29.22±3.23 months. Graft take rate was 93.4 % in all the cases, as well as 94.1%, 100%, and 85.7% in Group 1, Group 2, and Group 3, respectively. The mean values of ABG were improved from 35.17±6.64 to 23.52±10.4, 30.46±5.89 to 17.20±8.04, and 29.14±8.37 to 16.14±5.02 dB in Group 1, Group 2, and Group 3, respectively (P<0.05).

**Conclusion:**

Tri-layer tympanoplasty is a reliable procedure in the surgical treatment of the chronic otitis media with high-risk re-perforations.

## Introduction

The eradication of the middle ear disease, sound transmission mechanism, reconstruction from an intact tympanic membrane, and creation of air-containing middle ear space are mentioned as fundamental principles of tympanoplasty (1). During the last 70 years, many surgical techniques have been developed and employed successfully in an attempt to close perforations, and several materials, such as vein grafts, perichondrium, cartilage, fat, and homologous dura or fascia have been used as grafting materials (2,3). The grafting of temporalis fascia is the most frequently utilized approach for tympanic membrane perforations. However, cartilage is performing temporalis fascia as a grafting material, especially for repairing the scutum defects and large perforations to prevent or correct the failure of previous procedures associated with atelectatic tympanic membranes and chronic tubal dysfunction (4). In the atelectatic ears, the perichondrium and fascia have been shown to undergo atrophy and subsequent failure in the postoperative period (5-8). 

Cartilage might be a better alternative to resist the negative middle ear pressure that is observed in atelectatic ears (9). In addition to its stability to negative middle ear pressure, cartilage has more advantages, including less anti-inflammatory tissue reaction and well-incorporation with the tympanic membrane (4). However, it reduces the vibration pattern of the tympanic membrane contributing to some impairments in functional results. Nevertheless, according to the evidence, there was no significant difference between perichondrium and temporalis fascia in hearing gain (10,11); however, one might propose conductive hearing loss, especially in higher tones.

The results of recent studies have focused on cartilage reinforcement tympanoplasty in which temporal fascia is supported, especially in the aditus ad antrum region by placing the cartilage graft medially and posterosuperiorly to prevent the recurrences of retraction pockets or placing medially and anteriorly to prevent the medialization of fascial graft from the anterior portion of large perforations (12). 

According to the literature, the presence of some factors related to patient or disease itself forces the physician to classify the chronic ear disease as high-risk perforations. Those factors are subtotal, bilateral or total perforations, coexisting revision tympanoplasty, craniofacial abnormalities, cholesteatoma, and atelectatic ears (12,13). Many researchers are seeking a new surgical method for the treatment of those ears with a high success rate. The purpose of the present study was to present a different technique of cartilage tympanoplasty (i.e., tri-layer tympanoplasty) and its otological and audiological results in patients with high-risk perforation.

## Materials and Methods

**Study design**

Approval to perform the study was obtained from the Ethical Committee of our institute. This retrospective study was conducted on 42 out of 194 patients who underwent ear surgery in our tertiary department during 2008 and 2014. In a review of the patients’ chart, 42 patients (22 females and 20 males) were noted to undergo tri-layer tympanoplasty. Out of 42 cases, 4 subjects had bilateral tri-layer tympanoplasty, and then 46 ears were included in the present study. These patients experienced tri-layer tympanoplasty, which is described in detail in the surgical procedure section. The Institutional review board (IRB) was taken for the patients (Number: 66519339-900-01/2015/10-07). Informed written consent was obtained from the patients. All the authors equally contributed to the present project.

 All patients’ anamneses were noted, and detailed otorhinolaryngological examinations were performed. The examination of the external ear canal, any discharge and its character, as well as size and location of perforations, were recorded. All the patients underwent a systemic physical examination. All surgeries were carried out by three senior surgeons, and the patients were followed up at the third, sixth, and twelfth months in our clinic. After 2 years, all the subjects in this study were called by telephone and asked to come to the clinic for detailed autoscopic/endoscopic examination and audiological measurements. The patients were assigned into three groups, including grade 4 atelectasis according to Sade classification (Group 1) (14), total/near-total perforations (Group 2), and revision surgery (Group 3). Recently, the application of tri-layer tympanoplasty was initiated with intact canal procedure in the ears with cholesteatoma if it is ensured for the complete removal of cholesteatoma. However, those patients were not included in the present study in order to have a more homogenous group, and the follow-up period for those cases was short.

**Three groups**

Group 1 included grade 4 atelectasis of the tympanic membrane. The ossicular chain was reconstructed either by incus transposition/ interposition or a piece of the mastoid cortex. Titanium ossicular prosthesis was used when faced with the erosion of the stapes. 

Group 2 consisted of total/near-total total perforations of the tympanic membrane. All the patients in this group had an intact ossicular chain. The middle ear mucosa that was preferred to be dry was also operated and included in this group. Group 3 comprised revision patients with no history of cholesteatoma. Subtotal/large re-perforations were not included as revision cases. When necessary, atticotomy or antrotomy were performed in order to reach the posterior compartment of the retraction pocket. Mastoidectomy was not conducted if the retraction pocket could be removed by the aforementioned techniques. When the incus was eroded, the harvested cortical bone was utilized for ossicular reconstruction. All the subjects underwent intact canal-wall tri-layer tympanoplasty.

**Ear preparation**

For Group 1, the atelectatic eardrum was carefully elevated off the promontory without damaging the mucosa if possible. Ossicles reconstructed with bone cement were not included in this study. When faced with the erosion of the stapes, ossicular prosthesis was preferred for bone conduction, and those patients were not included in this study.

For Group 2, the tympanic membrane was pilled off from the malleus. Especially in total perforations, the tympanic rim may be totally anteriorly absent, and the anterior wall of the external auditory canal may have a smooth continuation to the mucosa of the Eustachian tube. In such a situation, a pocket for the graft replacement is performed by the retrograde elevation of the anterior epithelium of the external auditory canal as described in a study carried out by Farrior JB (15). 

For Group 3, since those patients had previous surgery, careful examination of the middle ear was essential for possible cholesteatoma. All the epithelial debris and remnant of the previous grafts were removed from the middle ear in order to have a fresh wound. 

**Replacement of fascia and cartilage graft**

Firstly, the temporalis fascia was placed laterally to the malleus and medially to the remnant tympanic membrane. The tympanomeatal flap and fascia graft were attached and anteriorly turned. Prepared cartilage was placed under the fascia and over the malleus. The notch on cartilage should sit on the malleus, and the perichondrium turned to the posterior bony canal. Then, the facia and flap were also laid posteriorly over the perichondrium as shown in Figure 1a. The cartilage graft should be large enough to occupy all the tympanic membrane. All the grafts and flaps were supported by the external auditory canal. When posterosuperior drilling was large enough to permit the postoperatively attic retraction, the attic was also reconstructed with the second piece of cartilage as shown in Figure 1b.

**Fig 1:**
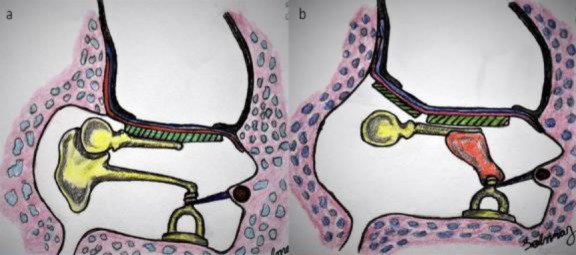
**A-** Facia and flap laying posteriorly over perichondrium **B-** Attic reconstruction with second piece of cartilage

**Preparation of grafts**

Temporalis fascia and cartilage graft were taken at the beginning of the surgery. Tragal cartilage was preferred since it is flat. The perichondrium was peeled on one side while attached to the graft and turned back to the posterior bony wall beneath the fascia and tympanomeatal flap. A small notch was superiorly made for the malleus without damaging the outer perichondrium as shown in Figure 1c.

**Fig 1:**
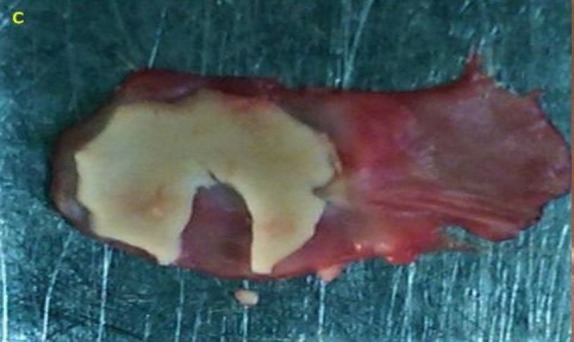
**c- **A small notch for malleus

**Outcome measurements**

All patients had preoperative audiograms and then were reviewed at 3 months, 6 months,12 months, and 2 years after the operation. The main outcome was successful graft take rate at 24 months, and after the surgery, the secondary outcome was the hearing gain. Other parameters were also documented, such as the retraction of the tympanic membrane and otorrhea. Since the optimal measurements of hearing outcomes were recommended in American Medical Association guidelines, audilogical results, pre- and post-operative air-bone gaps (ABG) for 0.5, 1, 2, and 3 kHz were calculated as decibels (16). Tympanic membrane retraction was also an important outcome of the present study. This was classified as retraction present of absent. Positivity was defined as the adhesion of the reconstructed membrane to the middle ear and promontory or presence of pocket due to the findings of endoscopic examination of the tympanic membrane at least 2 years after the surgery.

**Statistical Analysis**

The differences in ABG were assessed using the Student’s t-test. P-value of ≤0.05 was considered statistically significant.

## Results

The patients were within the age ranges of 16-59 years with an average of 35.09 years. All preoperative audiological and endoscopic findings of the ears were noted during the 1 week before the operation. The mean follow-up of all the cases was reported as 29.22±3.23 months. Group 1 consisted of 17 patients with grade 4 atelectatic tympanic membranes due to the Sade classification (14). Graft take was successful for 16 (94.1%) cases out of 17 patients at the end of at least 2 years. Two cases had atelectatic membrane to promontory but no perforation. Only one patient had anterior re-perforation, and this case had a history of postoperative ear discharge. 

Regarding audiological outcomes, the comparison of pre- and post-operative results revealed that mean air conduction level and mean ABG of four different frequencies in dB (500, 1000, 2000, and 3000 Hz) improved significantly from 44.29±5.27 to 32.94±7.72 and 35.17±6.64 to 23.52±10.4, respectively (P<.0001). Mean bone conduction was not statistically changed (P:0.88; Table.1).

**Table 1:** Pre-operative and post-operative audiological outcomes of grade 4 atelectatic ears

**Group 1** **N:17**	**Mean air conduction**	**Mean bone conduction**	**ABG**
Pre-operative	44.29±5.27	9.12±5.93	35.17±6.64
Post-operative	32.94±7.72	9.41±5.56	23.52±10.4
*P -value	<0.0001	0.88	<0.0005

* student t-test

Group 2 consisted of 15 subjects with total/ near-total tympanic membranes perforations. None of the cases had re-perforation at the end of 2 years. Graft take was reported as 100%. Regarding audiological outcomes, the comparison of pre- and post-operative results revealed that mean air conduction level and mean ABG of four different frequencies in dB (500, 1000, 2000, and 3000 Hz) improved significantly from 37.26±4.86 to 24.66±6.11 and 30.46±5.89 to 17.20±8.04, respectively (P<.0001). Mean bone conduction was not statistically changed (P:0.74; Table.2).

**Table 2:** Pre-operative and post-operative audiological outcomes of total/near-total perforations

**Group 2** **N:15**	**Mean air conduction**	**Mean bone conduction**	**ABG**
Pre-operative	37.26± 4.86	6.80±3.28	30.46±5.89
Post-operative	24.66± 6.11	7.20±3.21	17.20±8.04
*P- value	<0.0001	0.74	<0.0001

* student t-test

Group 3 consisted of the cases with 14 revision tympanoplasties. Out of 14 patients, 12 subjects (85.7%) had successful graft take. Two cases had re-perforation after revision surgery. Regarding audiological outcomes, the comparison of pre- and post-operative results revealed that mean air conduction level and mean ABG of four different frequencies in dB (500, 1000, 2000, and 3000 Hz) improved significantly from 43.21±5.24 to 30.28±6.48 and 29.14±8.37 to 16.14±5.02, respectively (P<.0001). Mean bone conduction was not statistically changed (P:0.97; Table.3).

**Table 3:** Pre-operative and post-operative audiological outcomes of revision tympanoplasty patients

**Group 3** **N:14**	**Mean air conduction**	**Mean bone conduction**	**ABG**
Pre-operative	43.21±5.24	14.78±4.61	29.14±8.37
Post-operative	30.28±6.48	14.85±4.19	16.14±5.02
*P value	<0.0001	0.97	<0.0001

* student t-test

Canal wall-up mastoidectomy was performed on 14 ears. Those ears had soft tissue images on temporal computed tomography scan but no evidence of cholesteatoma on microscopic examination during the surgery. Inside-out atticotomy was performed on 11 ears of atelectatic ears (Group 1). This manipulation was targeted to detect the present status of the ossicular chain and reconstruct it with incus transposition if necessary. Figure 1d shows a postoperative view of the tympanic membrane after tri-layer tympanoplasty.

**Fig 1:**
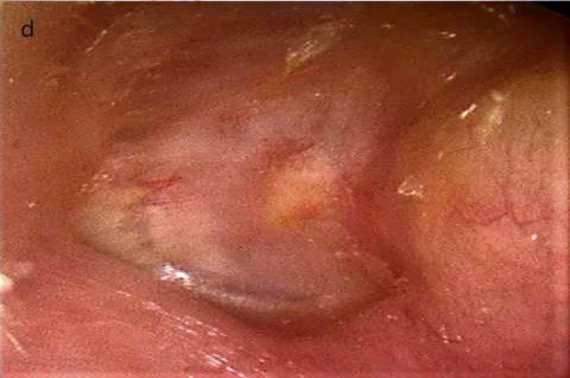
*D* ***Postoperative view of tympanic membrane after tri-layer tympanoplasty***

## Discussion

The temporalis fascia and tragal/conchal cartilages are the most common graft materials used in repairing perforated eardrum (12,13). The aim of the present study was to combine the temporalis fascia, as well as cartilage and its attached perichondrium (tri-layer), to have more stable new tympanic membrane in high-risk perforations. 

Low take rates of the tympanic membrane in some patients and studies are explained by the presence of some comorbid factors, such as retraction pockets, eustachian tube dysfunction, tympanic membrane atelectasis, extended perforations, and revision surgery. A review of the literature shows that although there is no general agreement and clear-cut definition, many otologists highlight the importance of those ears with such factors and named them high-risk perforations (12,15). For those patients, clinicians are forced to search for new graft materials and techniques.

The rigidity and thickness of cartilage raise questions about the functional results of cartilage grafts; however, there are many reports in the literature that have proven the similar rehabilitative effects of cartilage and fascia tympanoplasty on hearing outcome. The patients with cartilage tympanoplasty usually have more severe middle ear disorders, such as atelectasis, recurrent middle ear disease, or large perforations than those with temporalis fascia reconstruction (17-20). 

On the other hand, in a study conducted by Yetişer and Hıdır (4), this problem reduced because all the patients had primary tympanoplasty. It was concluded that the hearing gain in cases with cartilage grafting is much better than that in subjects with fascia tympanoplasty. Even if it is accepted that cartilage and fascia tympanoplasties have similar functional results on hearing outcome, cartilage has more advantages due to its stiffness (18).There are many techniques for cartilage tympanoplasty, such as Crowncork, inlay butterfly cartilage graft, composite autograft shield, cartilage palisade tympanoplasty,cartilage mosai ctympanoplasty, and perichondrium island flap (21-23). Recently a relatively new technique named cartilage reinforcement tympanoplasty was proposed by several otologists to prevent recurrent retractions and increase the take rate of the tympanic membrane in high-risk perforations (12). Cartilage has also been used in the reconstruction of the bony defect of the posterosuperior canal. 

In the presented technique in this study, all the tympanic membranes were reinforced by the perichondrium attached cartilage. Although some similar techniques can be observed in the literature, tri-layer tympanoplasty defined as the reinforcement of whole tympanic membrane by the cartilage and its perichondrium was firstly performed on the ears with high-risk perforation based on the present report.

In general, tympanoplasties are performed for two purposes, namely structural and functional outcomes. According to the results of numerous studies, it was shown that the take rate of tympanic membrane varying between 80% and 100% depends on the tympanoplasty technique and graft use. Although the cases in this study had high-risk perforations in which the take rate would be expected to be low, as 93.4%. Therefore, the first outcome of tri-layer tympanoplasty is that it is an effective method in the reconstruction of the tympanic membrane for the ears with high-risk perforations.

The second important outcome of ear surgery is the functional result, which means hearing recovery. Since the tympanic membrane in this technique is tri-layer and thick, it may be criticized due to the possible negative effect on vibration and sound transmission. In all three groups, postoperative ABG improved between 10 and 15 dB, compared to pre-operative values (P<0.05). 

The uncertainty is that if those ears were exclusively operated on either fascia or cartilage tympanoplasty, what would be postoperative ABG? A review of the literature can provide an answer to that question. Studies focused on the functional results of type I fascia and cartilage tympanoplasty documented the improvement of ABG less than 20 dB in many series (17-21). This finding shows even with the thick and tri-layer membrane, good hearing results can be achieved in the ears with high-risk perforations. In this study, Group 1 consisted of 17 atelectatic ears in which re-atelectasis was a serious problem after the surgery. Only two patients in this group had re-atelectatic membrane to promontory after 2 years of follow-up. Since the hearing results of these two subjects were acceptable, and there was no unseen pocket in the otoscopic examination, they were subjected to close follow-up and did not undergo revision surgery. 

Long-term follow-up is necessary to assess functional and anatomic outcomes in the atelectatic ears, since the recurrence of atelectasis may take several years to manifest. There is no study regarding the comparison of long-term re-atelectasis rate after cartilage tympanoplasty. Since all of the ears were at high risk of failure in this study, the results may favor the use of the tri-layer tympanoplasty technique in the atelectatic ears.

Revision tympanoplasty is another important issue in ear surgery. In revision tympanoplasty by temporalis fascia, tragal perichondrium or periosteum was reported a 86% graft take in a study conducted by Kaylie et al. (22). In the present study, the obtained rate (85.7%) was similar to the above-mentioned reported rate. A 91% graft take rate was reported by Djalilian in 35 revision patients using scar tissue as a grafting material (23). Veldman and Braunius reported a 90% graft take rate in 389 revision subjects (24). For primary and revision tympanoplasties, a graft take of 92% using butterfly cartilage inlay technique was reported by Ghanem et al. (25).

The take rate of the present study in revision tympanoplasty was 85.7%, which was slightly lower than the average rate according to the literature. This may be due to the small number of patients in the revision group. Single-layer techniques are known to pose difficulty in securing the graft material to the remnant tympanic rim or annulus. Medial fascia grafts may leave a residual perforation and tend to retract. In a study conducted by Farrior JB (15), sandwich graft tympanoplasty was proposed as a relatively new technique for those types of difficult tympanic perforations. Although the defined technique in the aforementioned study is highly reliable it is difficult to apply, especially in a case with the prominent anterior or posterior ear canal. Tri-layer tympanoplasty was preferred for the repair of a totally perforated eardrum. 

In the present study, a 100% take rate was reported and no anterior blunting in 15 patients. By this technique, cartilage and fascia are more stabilized with the level of tympanic membrane/annulus that provides better take rates and less blunting of the anterior annulus. The improvement of mean change in ABG was not significant between the study groups (P>0.05). This means tri-layer tympanoplasty had a similar positive effect on hearing recovery for groups. The present study had some limitations. Firstly, the study was retrospective and nonrandomized. Some patients were lost during the follow-up. Another important problem was that the number of cases in total and in each group were small to generalize the results. On the other hand, the first aim of the present study was to present tri-layer tympanoplasty.

## Conclusion

Tri-layer tympanoplasty is a reliable procedure for the ears with high-risk perforations. Atelectaic tympanic membranes, revision patients, and total/near total tympanic perforations can be treated by this technique with high take rate and acceptable audiological outcomes**.**
